# The effect of an app-based health intervention on somatic symptoms among employees of the DHL Group: a longitudinal pilot study

**DOI:** 10.1007/s00420-025-02145-8

**Published:** 2025-06-18

**Authors:** Naby May, Stefanie Kasten, Thomas Schwan, Matthias Scharle, Andreas Tautz, Stephan Letzel, Pavel Dietz

**Affiliations:** 1 Institute of Occupational, Social and Environmental Medicine, University Medical Centre of the Johannes Gutenberg University, Mainz, Germany; 2 Occupational Health and Safety, DHL Group, Bonn, Germany; 3 Corporate Health Management Germany, DHL Group, Bonn, Germany; 4 Armedo GmbH, Wuppertal, Germany; 5 Institute for Teachers’ Health at the Institute of Occupational, Social and Environmental Medicine, University Medical Centre of the Johannes Gutenberg University, Mainz, Germany

**Keywords:** App, Health intervention, Delivery service, PHQ-15, Somatization, Regression

## Abstract

**Background:**

Somatic symptoms significantly contribute to absenteeism and healthcare costs, particularly in physically demanding professions such as postal and delivery services. The FC-Fit Challenge, an app-based workplace health intervention, aims to promote healthier lifestyles through personalized feedback, social interaction, and professional guidance, targeting lifestyle changes and reducing somatic symptoms.

**Objectives:**

This study evaluated the effect of the FC-Fit Winterchallenge 2021/22 on somatic symptoms over time, considering sociodemographic and work-related differences, and identified predictors of somatic symptoms to inform workplace health strategies.

**Methods:**

A longitudinal design with three measurement points was employed: at the start of the intervention (T1), immediately post-intervention (T2), and three-month follow-up (T3). At T1, 497 participants completed the survey. Sociodemographic and work-related variables, health behaviors, and mental health indicators were analyzed. Multiple regression identified significant predictors of somatic symptoms.

**Results:**

Somatic symptoms significantly decreased post-intervention (T1: 7.0 ± 4.6 vs. T2: 5.9 ± 4.5, p < 0.001), with sustained effects at T3. Women, full-time employees, and administrative staff showed the most pronounced reductions. Predictors of higher somatic symptom severity included female gender, lower education, painkiller use, stress, and burnout, while high physical activity was associated with lower severity. Subgroup analysis revealed variability in intervention effectiveness based on sociodemographic and occupational factors.

**Conclusions:**

The FC-Fit Challenge is a promising, scalable tool for workplace health promotion. Tailoring interventions to specific employee profiles and addressing predictors like stress and burnout can optimize outcomes. Future studies should target underrepresented groups, such as delivery workers, and use randomized controlled designs to validate findings.

## Introduction

Somatic symptoms can include a wide range of physical complaints, such as palpitations, dizziness, gastrointestinal problems, fatigue, or musculoskeletal pain (Henningsen 2018). Factors that trigger these symptoms can be organic as well as psychological causes. In addition, they can be caused by unfavorable lifestyles, stressful working conditions or difficult life episodes (Henningsen 2018; Li et al. 2016; Barsky et al. 2001). Somatic symptoms were reported to be the most common reasons for illness-related incapacity among employees. For example, Kocalevent et al. (2013) showed that an off-balance energy system and pain (especially of the musculoskeletal system, headache, chest pain and abdominal pain) were the most frequent symptoms for sick leave. The study also showed that somatoform symptoms represent a heavy burden on the health care system and that they are often associated with a long duration of treatment (Kocalevent et al. 2013). Therefore, in addition to medical treatment costs, somatic symptoms cause high economic absenteeism costs. In this context, a report by the Federal Institute for Occupational Safety and Health showed that diseases of the musculoskeletal system and connective tissue (22.6% days of incapacity for work) were the most common disease-related diagnostic group in Germany in 2020, causing an estimated loss of gross value added due to incapacity for work of 35.2 billion euros (0.9% of gross national income) (BAuA 2022). Consequently, workplace health promotion as a measure of prevention and improvement of employee’s health is an important element for large companies in particular (Walter et al. 2019). This is also due to the comparatively low implementation costs in contrast to the costs of absence due to incapacity for work. In 2008, Kramer et al. reported the benefits of workplace health promotion (Kramer et al. 2008). Digital workplace health promotion in particular represents a relatively new market with high demand (Walter et al. 2019), which was further pushed by the COVID-19 pandemic. First studies indicate a benefit for health through digital workplace health promotion. However, the scientific database on this issue is under-researched, and it can be assumed that the acceptance of digital workplace health promotion differs according to sociodemographic characteristics (Howarth et al. 2018; Robroek et al. 2021). In this context, a systematic review of reviews confirmed the positive effect of workplace health promotion, particularly on the prevention of mental health and musculoskeletal disorders (Proper and van Oostrom 2019).

In a detailed analysis of the Federal Office for Goods Transport taking the data of the Absenteeism Report (German: ‘Fehlzeitenreport’) into account, it was highlighted that employees in postal and delivery services in particular recorded an above-average number of days of incapacity for work due to musculoskeletal disorders. However, it should be noted that the number of absenteeism days in this occupational group due to mental health issues increased proportionally in 2019, even though these did not show above-average values (Bundesamt für Güterverkehr 2021). The professional group of delivery services is experiencing steady growth. For example, in 2020, 247,639 people were employed under the job description of postal and delivery services in Germany, which was equivalent to a share of 0.75% of all employees subject to social insurance contributions (Grobe and Braun 2021). In the same year, due to the COVID-19 pandemic and changes in the ordering behavior of the general population, an increase in delivery volume of almost 11% was recorded, raising the total volume to 4.05 billion shipments (BIEK 2021). In Germany, the DHL Group (former Deutsche Post DHL Group) counts around 200,000 employees, which makes the company the largest postal service company in Europe. Analogous to the branch-specific view of days of incapacity for work, the DHL Group has a high sickness rate of more than 9%. This value is based on the last five years and quite as double as high as the values reported for the other German industry groups, which are around 4–5% (Deutsche Post DHL Group 2022; Grobe and Bessel 2022; Destatis 2022). The results of the systematic review indicate a clear association between common musculoskeletal disorders and mental health issues, such as depression, anxiety, and subjective mental well-being, particularly in adults (Heikkinen et al. 2019). Given the significant overlap between physical and psychological health, this finding highlights the importance of further analyzing mental health issues in populations suffering from musculoskeletal disorders.

Because of the branch-specific burdens and increases mental health issues, workplace health promotion is also an important element of the DHL Group’s health strategy. As part of this strategy, health challenges entitled “FC-Fit” are performed since 2019. These challenges, with the overall aim to foster lifestyle changes, are performed twice a year (one winter challenge and one summer challenge) and last for 4 weeks. Using a commercial app called ‘B2 Fit’, participants of these challenges get regular reminders and instructions on health-related topics. In addition, participants are enabled to perceive daily information about their own diet, physical activity, potential stressors, sleep, and similar health-related topics by answering questions directly in the app. Furthermore, these health challenges include the opportunity to share and post the actions and the progress in social-media groups (teams), which creates a kind of challenge (with colleagues). Moreover, the app offers the participants the opportunity to get in contact and discussion with health coaches or nutritionists.

Because of the increased days of incapacity for work in the entire professional group of postal and delivery services and the above-average sickness rate among employees of the Deutsche Post DHL Group, there is a need for scientific evaluation of health promoting efforts and the specific conditions that predict health and health promotion in this professional group. Understanding the conditions and factors predicting health, especially among the severely affected collective of delivery service employees, contributes to evidence-based planning of strategies and interventions, as effective programs must target factors related to health. In this context, previous studies confirmed the burdens on employees due to environmental factors, working and environmental conditions, exertion, pressure to perform and control, and shiftwork and working on weekends (Certa and Schröder 2020).

However, in the professional population of postal and delivery services, there is a large knowledge gap with regard to evidence-based health promotion efforts. Therefore, in collaboration with the DHL Group, the present study aimed to address this knowledge gap. The present paper refers to the outcome ‘somatic symptoms’ which, as stated above, leads to above-average incapacity for work, especially among employees in the postal and delivery services. Two aims are focused on here. The first aim is to investigate the effect of the app-based health intervention (FC-Fit challenge) on somatic symptoms and to evaluate whether potential effects of this intervention on somatic symptoms differ with regard to sociodemographic and work-related variables. The second aim is to investigate the potential predictors of somatic symptoms among employees of the DHL Group, which is the largest European representative of the postal and service branch and represents a little scientifically researched and yet growing occupational field.

## Method

### Study design and procedure

To address the above mentioned research aims, the ‘FC-Fit Winterchallenge 2021/22’ (just called ‘challenge’ in the further text) was process-oriented, externally monitored and scientifically evaluated. Therefore, a quantitative online questionnaire was administered to the participants prior to (T1), directly after (T2), and three months after (T3, follow-up) the four week challenge. In addition, employees had the opportunity to undergo a health check-up with the responsible company medical officer at T1 and T2. During this check-up, cardiovascular data, body measurements, and blood values such as glucose and blood lipids were assessed. However, the results of this check-up are not included in the present paper, which focuses on the results of the quantitative survey.

The start of the challenge was launched in December 2021 with a nationwide announcement to all employees of the DHL Group. The challenge was promoted using the companies’ internal newsletter, the website, and with posters that were placed at the company’s headquarters in Bonn. To participate in the challenge, employees had to register in the app. During this process, a personal ID was generated and sent to the employees of the DHL Group by e-mail. As part of the challenge, the app was used to increase health awareness. For this purpose, a points system was used to reward healthy eating, physical activity, and mindfulness.

The survey was performed using the open source software ‘LimeSurvey’. It could be accessed at all time points by using an ID, which was generated during initial registration in the app. Approval to perform the study was obtained by the ethical committee of the Medical Association of Rhineland-Palatinate (application number: 2021-16233). The study was performed in accordance with the Code of Ethics of the World Medical Association (Declaration of Helsinki) for experiments involving humans and the Ethical Principles and Guidelines for the Protection of Human Subjects of Research by the American Psychological Association (APA). A monetary donation to a charitable organization was used as an incentive to increase the likelihood of participation by triggering emotional responses, as recommended, for example, by Gendall and Healey (2010) and others (Reichel et al. 2021). Specifically, it was offered that if 1000 participants completed the survey, €1000 would be donated to the Children’s Cancer Aid Foundation (German: Stiftung Deutsche Kinderkrebshilfe).

**Fig. 1:**
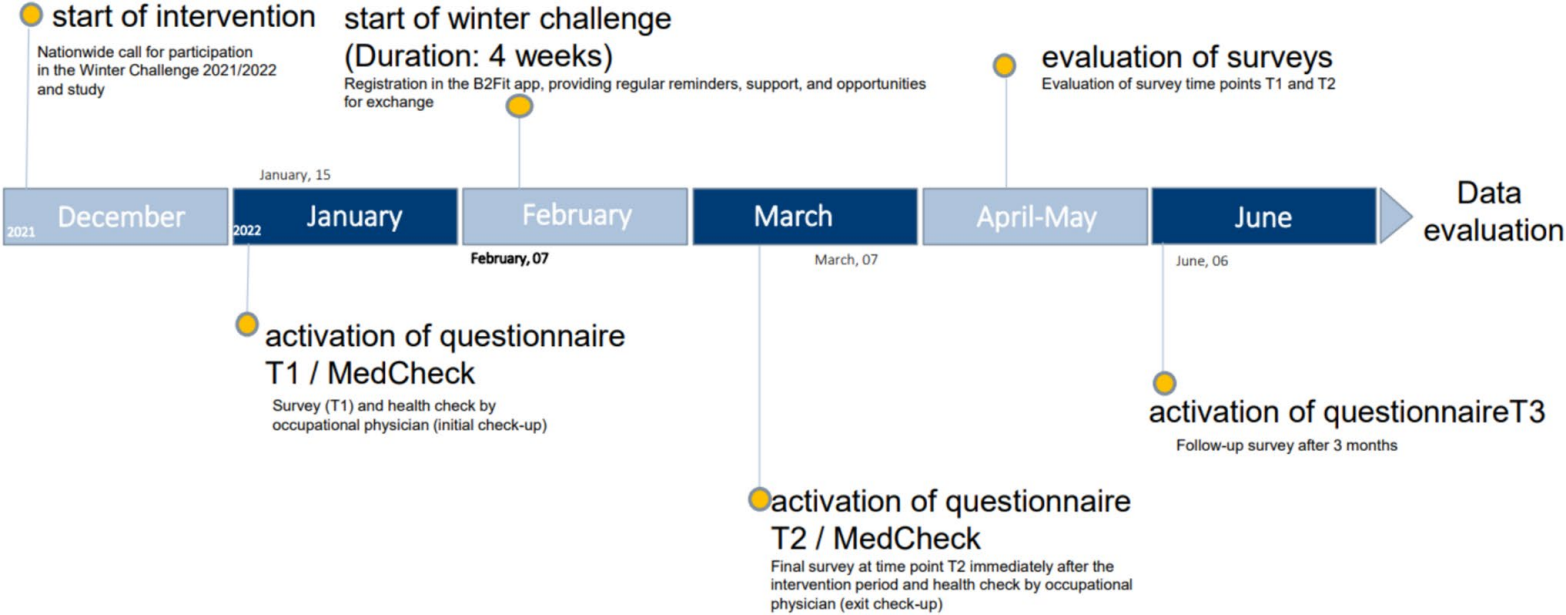
Timeline of the study

In the online survey, at T1, sociodemographic and work-related characteristics as well as various health-related constructs (described in detail below) were assessed. At T2 and T3, only the health-related constructs were further assessed to examine the effect of the challenge on health over time (Fig. 1).

### Measures

At T1, the survey included 45 questions. Whenever possible, established and validated measurement instruments were used. An overview of all questions used in the present survey is appended to this paper (Appendix 1). With regard to the first research aim, namely to investigate the effect of the challenge on somatic symptoms and to evaluate whether potential effects differ with regard to sociodemographic and work-related variables, the dependent variable somatic symptoms was assessed using the PHQ-15 questionnaire. It is a module for recording somatic symptoms as part of the Patient Health Questionnaire (PHQ) and suitable for measuring the severity of somatic symptoms and the severity of somatoform disorders. This is possible because no organic explanation of the symptoms is assumed for the evaluation (Kroenke et al. 2002; Stauder et al. 2021). This makes it useful in clinical practice as well as in the scientific consideration of mental and physical health. It includes the most common somatic symptoms (excluding upper respiratory symptoms) of outpatients, representing over 90% of reported physical complaints, and implements two additional questions on sleep disturbance and fatigue/energy loss. The self-report of the participants is possible on a scale of 0 (not bothered at all) to 2 (bothered a lot). In the scoring of the PHQ-15, values from 0 (no somatic symptoms) to 30 (severe somatic symptoms) can be obtained. In this context, values from 0 to 4 indicate minimal somatic symptoms. Values between 5 and 9 are interpreted as mild somatic symptoms, values between 10 and 14 are an indication of moderate somatic symptoms and scores of 15 and above are indicative for severe somatic symptoms (Kroenke et al. 2002).

In addition to sociodemographic and work-related variables, which are listed in Table 1 with their respective distributions and mean values for all three time points, the variable groups ‘health-related behavior’ and ‘mental health’ were also assessed. Referring to health-related behavior, the short form of the International Physical Activity Questionnaire (IPAQ-short) was used to measure physical activity (Sember et al. 2020) and the Alcohol Use Disorders Identification Test (AUDIT-C) to measure alcohol consumption. The AUDIT-C is an abridged version for the assessment of alcohol use disorders. It has been developed by the World Health Organization (WHO) and is one of the most common screening instruments for harmful and hazardous alcohol use worldwide (Babor and Robaina 2016). Additionally, the questions regarding cigarette and painkiller consumption were self-developed, with the response options adapted to the common vocabulary of scale items. For mental health variables, validated measurement instruments were used, including for burnout (COPSOQ-B13), anxiety and depression (PHQ-4), as well as stress (PSS-10). The COPSOQ-B13 is the burnout question module from the COPSOQ (Copenhagen Psychosocial Questionnaire). It is a scientifically validated questionnaire for the assessment of psychological resilience at work. In different studies, the quality criteria such as generalizability, construct validity or critical validity could be confirmed (Nübling et al. 2005). The PHQ-4 is an ultra-brief screening scale for anxiety and depression in the Patient Health Questionnaire (PHQ). It consists of the two questions from the PHQ-2, used to screen for depression, and the two questions from the GAD-2 (Generalized Anxiety Disorder), which is used to screen for general anxiety. The PHQ-4 has scientifically accepted construct validity and can be used as a general marker of psychological distress (Kroenke et al. 2009). The Perceived Stress Scale (PSS-10) is a widely used measurement scale for perceived stress. It is associated with depression, anxiety, lower life satisfaction, and lack of motivation. Here, the ability to cope with stress and the perceived excessive demands of everyday life will be questioned (Cohen et al. 1983; Klein et al. 2016).

**Table 1:** Sociodemographic and work-related characteristics of study samples

Variable	Baseline (T1)	Longitudinal sample (T2)	Follow-up (T3)
**Sociodemographics**			
Gender, No. (%)	497	133	96
Female	314 (63.2)	92 (69.2)	57 (59.4)
Male	183 (36.8)	41 (30.8)	39 (40.6)
Age, range in yrs (mean ± SD)	19–64 (44.45 ± 10.88)	24–64 (46.59 ± 10.34)	25–63 (47.76 ± 9.26)
Living form, No. (%)	491	131	95
Living alone	93 (18.9)	24 (18.3)	17 (17.9)
With partner	176 (35.8)	48 (36.6)	41 (43.2)
With partner and child(ren)	166 (33.8)	45 (34.4)	31 (32.6)
Only with child(ren)	37 (7.5)	11 (8.4)	5 (5.3)
With other person(s)	19 (3.9)	3 (2.3)	1 (1.1)
Educational degree, No. (%)	493	133	95
Without educational qualification	1 (0.2)	1 (0.8)	1 (1.1)
Secondary school	38 (7.7)	14 (10.5)	12 (12.6)
Intermediate secondary school	131 (26.6)	35 (26.3)	25 (26.3)
University of applied sciences entrance qualification	31 (6.3)	9 (6.8)	6 (6.3)
General university entrance qualification656	65 (13.1)	13 (9.8)	10 (10.5)
Bachelor degree	94 (19.1)	30 (22.6)	16 (16.8)
Master degree	129 (26.2)	30 (22.6)	24 (25.3)
Other degree	4 (0.8)	1 (0.8)	1 (1.1)
**Work-related characteristics**			
Work area, No. (%)	492	133	94
Administration	365 (74.2)	93 (69.9)	62 (66.0)
Mail or parcel center	31 (6.3)	10 (7.5)	8 (8.5)
Delivery	56 (11.4)	23 (17.3)	18 (19.1)
Transport	6 (1.2)	1 (0.8)	1 (1.1)
Other area	34 (6.9)	6 (4.5)	5 (5.3)
Leading function, No. (%)	494	131	95
No	343 (69.4)	101 (77.1)	72 (75.8)
Yes, without personnel responsibility	69 (14.0)	14 (10.7)	10 (10.5)
Yes, with personnel responsibility	82 (16.6)	16 (12.2)	13 (13.7)
Working time model, No. (%)	493	131	94
Full-time	391 (79.3)	96 (73.3)	68 (72.3)
Part-time	102 (20.7)	35 (26.7)	26 (27.7)
Type of Job, No. (%)	495	133	95
Predominantly mental	406 (82.0)	99 (74.4)	68 (71.6)
Predominantly physical	43 (8.7)	18 (13.5)	13 (13.7)
Equally mental and physical	46 (9.3)	16 (12.0)	14 (14.7)

### Statistical analysis

The description of the statistical analyses will be divided into two parts, based on the two study aims.

With regard to the first study aim, namely to investigate the effect of the challenge on somatic symptoms and to evaluate whether potential effects differ with regard to sociodemographic and work-related variables, descriptive results are presented as means with standard deviations (SD). To compare values between T1 and T2 as well as T2 and T3, paired t-tests were used if the conditions that apply for this analysis were fulfilled. Results are presented with T-values, effect sizes (d) and p-values (p). To investigate potential differences in means between subgroups, paired t-tests were performed if there were two values in the relevant subgroup, the variables were normally distributed, or had a minimum of 30 samples in each value. When these conditions could not be met, Mann–Whitney-U-test (U) with Z-statistic (Z) was used for the analysis of these subgroup comparisons. For variables with more than two values, Kruskal–Wallis-test (X^2^) was used. When considering the longitudinal differences for each subgroup, Wilcoxon-Signed-Rank-test (z) with the effect size (r) was applied. All analyses were checked additionally with parametric tests in order to secure the results.

To address the second aim of the study, namely to investigate potential predictors of somatic symptoms among employees of the Deutsche Post DHL Group, a linear multiple regression analysis was performed with the data of T1 using the PHQ-15 score as dependent variable. The T1 sample was chosen for the multiple linear regression because at T1, the sample size was sufficient compared to T2 and T3. With a general population of around 225,000 employees of the Deutsche Post DHL Group in Germany, a priori power analysis revealed a sample size of at least 384 cases for the regression analysis. A confidence level of 95% and a margin of error of 5% were assumed.

The sociodemographic independent variables were included in the regression analysis as follows: *Age* was treated as a metric variable but not centered at the mean, as the study registration criteria already specified that participants had to be adults, and the maximum age was capped at 65 years. With regard to the binary variable *gender*, ‘male’ is the reference category. The variable *educational degree* was recoded to a dummy variable representing a binary variable having the general qualification for University entrance or not. Here, the category ‘no’ is the reference category. With regard to the work-related independent variables, the variable *working time model* was divided into full-time and part-time employment, with part-time employment as the reference category. The variable *work area* was recoded as a dummy variable. It is now indicating if the participants work in the administration sector or not with the reference category ‘no’. The recoding into dummy variables was necessary because a comparison between the subgroups was not possible due to strongly differing case numbers. Finally, with regard to the variable *type of job*, the category ‘predominantly mental’ was used as a reference, contrasted with the categories ‘predominantly physical’ and ‘equally mental and physical’. Referring to variables on health-related behavior, the variables *alcohol, cigarette* and *painkiller consumption* were recoded into dummy variables, with the category ‘no’ being used as the reference for each variable. The variable *physical activity* was divided into the categories ‘insufficiently active’, ‘moderately physically active’, and ‘highly physically active’, with insufficiently activity being the reference category in each case. The independent variables associated with mental health were integrated for the regression analysis as follows: The Perceived *Stress Score (PSS-10)* and the *Burnout Score (COPSOQ B13)* were used as metric scaled variables. *Depression (PHQ-2)* and Generalized *Anxiety Disorder (GAD-2)* were recoded into dummy variables according to their specific cut-off values. The negative screenings are considered the reference category in each case. The test for multicollinearity, conducted through a correlation matrix (Urban and Mayerl 2018), variance inflation analysis, and the calculation of VIF values, indicates that the independent variables used are not exhibiting linear dependency on each other. In the residual analysis for homoscedasticity both the significant White test and the significant Breusch-Pagan/Cook-Weinsberg test suggest a violation of the homoscedasticity assumption, indicating heteroscedasticity. However, in such instances, the standard errors of the coefficients may become biased, potentially leading to erroneous assumption of significant effects. To address this, bootstrapping can be employed to mitigate heteroscedasticity. For this reason, bootstrapping with 1000 samples was performed in the regression analysis. Testing for normal distribution of the residuals, the histogram and PP-plot show an approximate normal distribution. The skewness and kurtosis of the studentized residuals were smaller than the cut-off ranges reported in the literature (MRC Cognition and Brain Sciences Unit 2018), therefore, normal distribution could be assumed. The statistical analyses were performed using IBM SPSS version 27.

In addition, mean comparisons were performed between T1 and T2 as well as T2 and T3 for the independent health-related variables that had significant regression results. These were performed using a paired t-test (t), analogous to the treatment of the first aim of the study, if the precondition test was met (t, d, p). If the precondition test was not fulfilled, the results could be tested with the nonparametric Wilcoxon-Signed-Rank-test (z, r, p).

## Results

### Sample characteristics

The total case number at the different time points decreased from T1 to T3. At T1, N = 497 participants entered the analysis, at T2, N = 133 participants, and at T3, N = 96 participants. The distribution of the sociodemographic and work-related variables at the three time points is presented in Table 1. In short, around two-thirds of the sample were women (T1: n = 314; 63.2%, T2: n = 92; 69.2%, T3: n = 57; 59.4%). The mean age at the three time points ranged between 44 and 48 years, and most of the participants (n = 288; 58.4%) had at least the university entrance qualification. With regard to work area, over two-thirds (T1: n = 365; 74.2%, T2: n = 93; 69.9%, n = 62; T3: 66.0%) of the participants worked in an administrative area, and also over two-thirds had full-time employment (T1: n = 391; 79.3%, T2: n = 96; 73.3%, T3: n = 68; 72.3%).^1^ Lastly, most of the participants engaged in predominantly mental work activities (T1: n = 406; 82.0%, T2: n = 99; 74.4%, T3: n = 68; 71.6%).

### Somatic symptoms over time and between subgroups

The mean value for somatic symptoms at T1 (before the challenge) was 7.0 ± 4.6. This value decreased significantly (p < 0.001) to 5.9 ± 4.5 at T2 (directly after the challenge), with a small effect size (t = 4.15, d = 0.38). The 3-month follow-up analysis (T3) revealed a mean value of somatic symptoms of 5.7 ± 4.3. Compared to T2 (5.3 ± 4.2), this small increase was not significantly different (t = −1.21, d = n.s., p = 0.230), see Table 2.

**Table 2:** Means, pre-to-post and post-to-follow-up comparison of somatic symptoms

Variable	T1	T2	N, T^a^, d, p	T2	T3	N, T^a^, d, p
	Mean ± SD			Mean ± SD		
Somatic symptoms	7.0 ± 4.6	5.9 ± 4.5	N = 119, t = 4.15, d = 0.38***	5.3 ± 4.2	5.7 ± 4.3	N = 62, t = −1.21, d = n.s

^a^ = paired t test (t, d, p); p < 0.05*, p < 0.01**, p < 0.001***

The metrics of the two comparisons are not directly comparable due to differing sample sizes

With regard to sociodemographic and work-related group differences of somatic symptoms (Table 3), gender-specific analysis at T1 revealed that females (7.7 ± 4.7; n = 87) showed significantly higher (p = 0.005) values of somatic symptoms compared to males (5.3 ± 3.8; n = 32) with a medium to large effect size (t = −2.91, d = −0.71). At T2, this gender-specific difference was observed as well but with a rather small effect (t = −2.35, d = −0.44). Finally, longitudinal analysis with regard to gender indicates a statistically significant (p < 0.001) decrease of somatic symptoms from T1 to T2 among females only with a medium effect size (z = −3.48, r = 0.38). Analyzing work-related group differences in somatic symptoms, no significant subgroup differences were revealed, neither at T1 nor at T2. However, when considering longitudinal differences, it can be observed that for different work areas (N = 119), only the ‘*Administration*’ subgroup (n = 86) showed a significant reduction in somatic symptoms from T1 (6.7 ± 4.6) to T2 (5.4 ± 4.0), with a moderate effect size (z = −3.67, r = 0.40, p < 0.001). Regarding the variable *working time model* (N = 117), full-time employees (n = 85) demonstrated a significant decrease in symptoms from T1 (7.0 ± 4.9) to T2 (5.7 ± 4.7), also with a moderate effect size (z = −3.79, r = 0.41, p < 0.001). Furthermore, in analyzing the variable *type of job* (N = 119), a significant reduction in somatic symptoms from T1 to T2 was observed exclusively for the ‘predominantly mental’ subgroup (n = 90), with somatic symptoms decreasing from 6.7 ± 4.5 at T1 to 5.4 ± 4.0 at T2, demonstrating a moderate effect (z = −3.85, r = 0.41, p < 0.001).

**Table 3:** Means and subgroup-comparisons of somatic symptoms pre-to-post (T1 to T2)

Variable	T1		T2		Longitudinal difference
	Mean ± SD	Subgroup differences	Mean ± SD	Subgroup differences	
Gender, N = 119		t^a^ = −2.91, d = −0.71**		t^a^ = −2.35, d = −0.44*	
Female, n = 87	7.7 ± 4.7		6.5 ± 4.8		z^b^ = −3.48, r = 0.38***
Male, n = 32	5.3 ± 3.8		4.3 ± 3.0		z^b^ = −1.64, r = n.s
Work area, N = 119		X^2c^ = 3.18, n.s		X^2c^ = 6.21, n.s	
Administration, n = 86	6.7 ± 4.6		5.4 ± 4.0		z^b^ = −3.67, r = 0.40***
Mail/parcel center, n = 10	7.4 ± 3.8		6.0 ± 4.2		z^b^= −1.07, r = n.s
Delivery, n = 19	8.7 ± 5.2		8.4 ± 6.1		z^b^ = −0.44, r = n.s
Transport	n.a		n.a		n.a
Other area, n = 4	6.0 ± 2.5		4.5 ± 4.7		z^b^ = −1.07, r = n.s
Working time model, N = 117		t^a^ = 0.343, d = n.s		t^a^ = 1.223, d = n.s	
Full-time, n = 85	7.0 ± 4.9		5.7 ± 4.7		z^b^= −3.79, r = 0.41***
Part-time, n = 32	7.3 ± 3.7		6.7 ± 3.8		z^b^ = −1.10, r = n.s
Type of job, N = 119		X^2c^ = 3.07, n.s		X^2c^ = 5.11, n.s	
Predominantly mental, n = 90	6.7 ± 4.5		5.4 ± 4.0		z^b^ = −3.85, r = 0.41***
Predominantly physical, n = 16	8.1 ± 3.5		7.3 ± 3.6		z^b^ = −0.98, r = n.s
Equally mental and physical, n = 13	8.4 ± 6.1		7.8 ± 7.4		z^b^ = −0.48, r = n.s

^a^ = paired t-test (t, d, p), ^b^ = Wilcoxon-signed-rank-test (z, r, p), ^c^ = Kruskal–Wallis-test (X^2^, p), p < 0.05*, p < 0.01**, p < 0.001***

In order to test whether the effects of the challenge can be replicated over the long term, follow-up analysis (comparison between T2 and T3) was performed, which are presented in Table 4. It shows that no significant differences between T2 and T3 were observed.

**Table 4:** Means and subgroup-comparisons of somatic symptoms post-to-follow-up

Variable	T2		T3		Longitudinal difference
	Mean ± SD	Subgroup differences	Mean ± SD	Subgroup differences	
Gender, N = 62		U^a^ = 386.5, Z = −0.657, n.s		t^b^ = −1.93, d = n.s	
Female, n = 41	5.7 ± 4.5		6.4 ± 4.6		z^c^ = −1.74, r = n.s
Male, n = 21	4.7 ± 3.4		4.4 ± 3.5		z^c^ = −1.06, r = n.s
Work area, N = 62		X^2 d^ = 3.26, n.s		X^2 d^ = 7.45, n.s	
Administration, n = 42	5.0 ± 4.2		5.4 ± 4.1		z^c^ = −0.61, r = n.s
Mail/parcel center, n = 5	5.8 ± 5.1		4.6 ± 3.1		z^c^ = −0.92, r = n.s
Delivery, n = 12	6.7 ± 3.9		8.3 ± 4.9		z^c^ = −1.98, r = n.s
Transport	n.a		n.a		n.a
Other area, n = 3	3.3 ± 4.9		1.7 ± 2.1		z^c^ = −1.00, r = n.s
Working time model, n = 61		U^a^ = 321.0, Z = −1.048, n.s		U^a^ = 298.0, Z = −1.414, n.s	
Full-time, n = 43	5.2 ± 4.3		5.3 ± 4.3		z^c^ = −0.12, r = n.s
Part-time, n = 18	5.9 ± 3.9		6.8 ± 4.4		z^c^ = −1.38, r = n.s
Type of job, N = 62		X^2 d^ = 3.62, n.s		X^2 d^ = 3.74, n.s	
Predominantly mental, n = 45	4.9 ± 4.2		5.2 ± 4.1		z^c^ = −0.34, r = n.s
Predominantly physical, n = 10	7.1 ± 3.2		7.4 ± 3.1		z^c^ = −0.54, r = n.s
Equally mental and physical, n = 7	5.4 ± 5.3		6.9 ± 6.6		z^c^= −1.38, r = n.s

^a^ = Mann–Whitney-U-Test (U, Z, p), ^b^ = paired t-test (t, d, p), ^c^ = Wilcoxon-signed-rank-test (z, r, p), ^d^ = Kruskal–Wallis-test (X^2^, p), p < 0.05*, p < 0.01**, p < 0.001***

### Predictors of somatic symptoms assessed by multiple linear regression analysis

Of the 16 independent variables, multiple linear regression analysis with bootstrapping revealed that six variables significantly predicted somatic symptoms (Table 5). With regard to sociodemographic variables, female gender (standardized β = 0.117, p = 0.007) and lack of university access (standardized β = −0.128, p = 0.004) positively predicted somatic symptoms. Regarding health-related behaviors, the variable consumption of painkillers also positively predicted somatic symptoms (standardized β = 0.126, p = 0.003). Conversely, high physical activity negatively predicted somatic symptoms (standardized β = −0.102, p = 0.027). Furthermore, within the mental health-related variables, both an increased stress score (standardized β = 0.132, p = 0.039) as well as a higher burnout score positively predicted somatic symptoms (standardized β = 0.395, p < 0.001). The explained variance in the overall model was 41.6% (adjusted R^2^).

**Table 5:** Predictors of somatic symptoms measured by patient health questionnaire (PHQ-15) in a multiple linear regression analysis with inclusion of the four variable groups: sociodemographic variables, work-related variables, variables for health-related behavior and mental health variables

Predictor	‘Reference category’	b (95% CI)^a^	SE_b_^a^	BETA	t	p
Const		−0.035 (−2.921, 2.688)	1.375		−0.024	0.980
**Sociodemographic variables**						
Age		0.006 (−0.025, 0.036)	0.016	0.015	0.356	0.722
Gender	‘Male’	1.067 (0.351, 1.802)	0.390	0.117	2.695	**0.007**
University access	‘No’	−1.134 (−1.954, – 0.315)	0.369	−0.128	−2.918	**0.004**
**Work-related variables**						
Working time model	‘Part-time’	0.518 (−0.405, 1.534)	0.478	0.048	1.053	0.293
Administration area	‘No’	0.308 (−0.844, 1.586)	0.629	0.031	0.557	0.578
Mainly physical work	‘Predominantly mental’	0.846 (−0.633, 2.397)	0.779	0.055	1.014	0.311
Equally mental and physical work	‘Predominantly mental’	0.686 (−0.948, 2.676)	0.940	0.046	0.922	0.357
**Variables for health-related behavior**						
Drink alcohol	‘No’	−0.088 (−0.787, 0.599)	0.347	−0.010	−0.239	0.811
Smoke cigarettes	‘No’	−0.835 (−1.841, 0.180)	0.490	−0.070	−1.665	0.097
Take painkiller	‘No’	1.102 (0.378, 1.816)	0.360	0.126	3.042	**0.003**
Moderate physical activity	‘Insufficiently active’	−0.109 (−1.242, 1.136)	0.578	−0.009	−0.199	0.842
Highly physical activity	‘Insufficiently active’	−0.887 (−1.546, −0.136)	0.377	−0.102	−2.223	**0.027**
**Mental health variables**						
Stress-score (PSS-10)		0.087 (0.009, 0.163)	0.040	0.132	2.075	**0.039**
Depression (PHQ-2)	‘Negative depressionsscreening’	1.344 (−0.231, 2.848)	0.771	0.100	2.130	0.034^b^
Anxiety disorder (GAD-2)	‘Negative anxiety screening’	0.872 (−0.573, 2.313)	0.701	0.067	1.397	0.163
Burnout-score (COPSOQ B13)		0.077 (0.054–0.099)	0.011	0.395	6.752	** < 0.001**
Adj. R^2^ = 0.416 N = 374						

Bold values indicate statistical significance

^a^Confidence intervals and standard deviations by BCa-bootstapping with 1000 BCa-samples

^b^The result for depression was not confirmed by bootstrapping. The confidence interval contains a change of the sign, so depression has no significant effect to PHQ-15

## Discussion

The present study investigated the impact of the ‘FC-Fit Winterchallenge 2021/22’ app-based health intervention on somatic symptoms among DHL Group employees. The study aimed to determine whether the intervention would reduce somatic symptoms and whether these effects varied by sociodemographic and work-related factors. Another aim was to identify predictors of somatic symptoms, particularly focusing on sociodemographic, work-related, and health-related factors.

Regarding the first study aim, the results show a significant decrease in somatic symptoms, particularly among women, full-time employees, and those in administrative roles. A notable result of this study is the gender difference in response to the intervention. Women, who initially reported higher levels of somatic symptoms, showed a more significant reduction than men. This difference aligns with previous findings that women may be more likely to report physical complaints, potentially due to heightened stress sensitivity or different coping mechanisms (Barsky et al. 2001; Piccinelli and Simon 1997). With regard to work-related factors, employees in administrative roles showed a substantial improvement in somatic symptoms. However, it should be noted that this study primarily included administrative staff, which limits the generalizability of the findings to other groups, particularly delivery workers, who represent the largest occupational group at DHL Group. Given the physical and psychological demands of delivery work, future research should aim to include a more representative sample from this group to understand better how digital health interventions affect their health outcomes.

Regarding the second study aim, namely to identify predictors of somatic symptoms, results reveal that several factors, such as female gender, lower education levels, higher stress, and burnout, were significantly associated with increased somatic symptoms. On the other hand, higher physical activity was associated with lower symptom levels. These findings suggest that sociodemographic and psychological factors play an essential role in predicting somatic symptoms. The strong association between burnout and somatic symptoms, in particular, is consistent with existing research (Kocalevent et al. 2013; Gavurova et al. 2022; Nordin et al. 2024) and highlights the close link between mental and physical health. Furthermore, the positive impact of physical activity on reducing somatic symptoms underscores the potential of promoting exercise within workplace health programs (Brüchle et al. 2021; Dahlstrand et al. 2021).

From a practical perspective, these findings suggest that app-based health interventions, such as the ‘FC-Fit Challenge’, can be an effective and cost-efficient way to improve employee health and reduce absenteeism due to illness. However, since the majority of participants in this study were from administrative positions, the effectiveness of such interventions for delivery workers remains unclear. Given the high physical demands of delivery work, it would be beneficial to adapt future interventions to better meet the needs of this key group within the company. However, this study has a few limitations that should be discussed. The reduction in sample size during the study leads to biases, which also limit the generalizability of the results. A notable limitation of this study is the insufficient statistical power of the subgroup analyses. Given the relatively small sample sizes within certain subgroups, these analyses may be underpowered. This limitation should be carefully considered when assessing the validity of the findings within these subgroups. Additionally, the use of self-reported data to assess somatic symptoms could introduce subjective biases. Another limitation is that the sample mainly consisted of employees from the administrative sector, while the largest group of employees at DHL Group are delivery workers. Consequently, the results may only partially capture the intervention’s impact across the entire workforce. Another limitation concerns the limited documentation of the digital intervention. No screenshots or detailed visual documentation of the B2 Fit app interface are available, and no usage data (e.g., login frequency or adherence rates) were collected. This restricts the ability to evaluate the intervention’s fidelity and limits reproducibility. Finally, a major limitation of the study design is the absence of a control group, which limits the ability to make definitive conclusions about the causal effects of the intervention. This reduces the internal validity of the study and should be carefully considered when interpreting the results. One potential approach to address the absence of a control group is the use of propensity score matching with non-participant data, which can help approximate counterfactual outcomes. However, we do not have access to non-participant data for this study, limiting our ability to apply this method. As a result, we are unable to estimate counterfactual outcomes, and this remains a notable limitation when interpreting the causal effects of the intervention.

Future research should aim to address these limitations by including more representative samples, especially from the delivery sector, and incorporating randomized controlled trials to assess the effectiveness of app-based health interventions better. We recommend incorporating a control group to strengthen the ability to draw causal inferences about the intervention’s effectiveness. Additionally, systematically monitoring app usage and collecting detailed engagement data would allow for a more thorough assessment of how usage patterns influence outcomes. This would help mitigate potential confounding factors, such as seasonal effects, and provide more robust evidence regarding the effectiveness of similar interventions. Additionally, further exploration of the gender-specific differences observed in this study, as well as the role of psychological factors such as stress and burnout, would be valuable. Moreover, investigating the long-term effects of such interventions on both physical and mental health outcomes in high-stress occupations should be a priority for future studies.

## Data Availability

The raw data supporting the results of the present article will be made available by the authors without undue reservation.

## Appendix 1: Questionnaire (original version)

Fragebogen zur FC fit Gesundheits-Challenge 2022.

## Footnotes

1 As the sample primarily includes administrative staff, the results cannot be generalized to other occupational groups such as delivery workers.
